# Effects of Amiodarone and *N*-desethylamiodarone on Cardiac Voltage-Gated Sodium Channels

**DOI:** 10.3389/fphar.2016.00039

**Published:** 2016-03-01

**Authors:** Mohammad-Reza Ghovanloo, Mena Abdelsayed, Peter C. Ruben

**Affiliations:** Department of Biomedical Physiology and Kinesiology, Simon Fraser University, BurnabyBC, Canada

**Keywords:** amiodarone, *N*-desethylamiodarone, electrophysiology, long QT, Nav1.5

## Abstract

Amiodarone (AMD) is a potent antiarrhythmic drug with high efficacy for treating atrial fibrillation and tachycardia. The pharmacologic profile of AMD is complex. AMD possesses biophysical characteristics of all of class I, II, III, and IV agents. Despite its adverse side effects, AMD remains the most commonly prescribed antiarrhythmic drug. AMD was described to prolong the QT interval and can lead to torsades de pointes. Our goal was to study the effects of AMD on peak and late sodium currents (I_Na,P_ and I_Na,L_) and determine whether these effects change as AMD is metabolized into *N*-desethylamiodarone (DES). We hypothesized that AMD and DES block both I_Na,P_ and I_Na,L_ with similar profiles due to structural similarities. Given the inherent small amounts of I_Na,L_ in Na_V_1.5, we screened AMD and DES against the Long QT-3-causing mutation, ΔKPQ, to better detect any drug-mediated effect on I_Na,L_. Our results show that AMD and DES do not affect WT or ΔKPQ activation; however, both drugs altered the apparent valence of steady-state fast-inactivation. In addition, AMD and DES preferentially block ΔKPQ peak conductance compared to WT. Both compounds significantly increase I_Na,L_ and window currents. We conclude that both compounds have pro-arrhythmic effects on Na_V_1.5, especially ΔKPQ; however, DES seems to have a greater pro-arrhythmic effect than AMD.

## Introduction

In the 1960s, an iodine-containing benzofuran compound called amiodarone (AMD) was developed as a therapeutic vasodilator (Phillips and Bauman, 1995). Decades of research and clinical trials have shown the effects of AMD in a range of organ systems. AMD slowly became a widely used antiarrhythmic drug, with a high efficacy for treating conditions including atrial fibrillation and tachycardia (Pollak, 1998). The pharmacologic profile of this drug is complex. Although AMD has been classified primarily as a class III antiarrhythmic drug, it also has biophysical characteristics of class I, II, and IV agents in that it blocks L-type calcium, potassium, and sodium currents (Singh and Vaughan Williams, 1970; Kodama et al., 1997; Nattel and Singh, 1999; Wu et al., 2008). AMD is the most commonly prescribed antiarrhythmic drug despite its potentially serious side effects, including adverse effects on thyroid glands, the pulmonary system, and the liver (Danzi and Klein, 2015).

In experimental cardiac preparations, the use of AMD is associated with prolongation of the QT interval and action potential duration (APD). These events may lead to torsades de pointes (Tarapues et al., 2014). AMD has a higher selectivity for the potassium and sodium channels that are expressed in atrial as opposed to ventricular myocytes (Suzuki et al., 2013). The atrial selectivity of sodium channel block may contribute to the treatment of atrial fibrillation (Antzelevitch and Burashnikov, 2009; Burashnikov and Antzelevitch, 2013). However, AMD’s potency to prolong the QT interval has led to its prohibition in patients with Long-QT Syndrome (Digby et al., 2011; Franz et al., 2014).

Long-term treatments with AMD is suggested to induce remodeling of ion-channel expression in a dose-dependent manner, leading to electrophysiological variations. Therefore, in addition to directly affecting membrane proteins, AMD also modifies the levels of ion-channel transcripts (Le Bouter et al., 2004).

Although several Cytochrome P450 (CYP) subtypes may contribute to metabolism of any given compound, the primary enzymes involved in the AMD metabolism are CYP3A4 and CYP2C8 (Ohyama et al., 2000; Zahno et al., 2011). AMD has a relatively long half-life of 40–50 days (Zipes et al., 1984). The full metabolism of AMD gives rise to 22 phase I and 11 phase II products (Deng et al., 2011). AMD’s most prominent metabolite is produced as a result of a *N*-deethylation reaction catalyzed by CYP3A4, producing DES, which is a pharmacologically active compound (Deng et al., 2011).

In a classic study conducted by Pallandi and Campbell (1987) on guinea-pig ventricular myocardium, it was determined that at clinically relevant levels, AMD and DES exhibit both class I and class III effects. The class I effects (on sodium channels) are rate dependent, which in conjunction with the class III effects (on potassium channels) may increase APD and reduce V_max_ (Pallandi and Campbell, 1987).

Despite the potency of AMD as an antiarrhythmic agent and the already existing body of knowledge, its effects on sodium channels are still poorly understood. We sought to study the effects of AMD on peak and late sodium currents (I_Na,P_ and I_Na,L_), and determine whether these effects change as AMD metabolizes into DES. We hypothesized that both AMD and DES block I_Na,P_ and I_Na,L_ with similar profiles due to their structural similarities. Given the low amplitude of I_Na,L_ in Na_v_1.5, we used the LQT-3 mutation, ΔKPQ, to increase I_Na,L_ and thus better detect potential drug-mediated effect on I_Na,L_. The mutant phenotype associated with ΔKPQ is manifested in the presence of large I_Na,L_ due to the deletion of three amino acids (Lysine, Proline, Glutamine) in the linker region between domains III and IV in Na_V_1.5 (Wang et al., 1995). This is a hot-spot region in Na_V_1.5 since it elicits fast-inactivation (Chandra et al., 1998). We report that AMD and DES have similar effects on the voltage-dependence of fast-inactivation and the amplitude of I_Na,L_, and can therefore contribute to pro-arrhythmic effects.

## Materials and Methods

### Cell Culture

Chinese hamster ovary (CHO) cells were transiently transfected with cDNA encoding either wild-type or the ΔKPQ mutant form of Na_V_1.5. Transfection was done according to the PolyFect transfection protocol. After each set of transfections, a minimum of 8 h incubation was allowed before platting. Dr. Robert Kass generously provided *SCN5A* cDNA encoding the ΔKPQ mutation.

### Electrophysiology

Whole-cell patch clamp recordings were performed in an extracellular solution containing (in mM): 140 NaCl, 4 KCl, 2 CaCl_2_, 1 MgCl_2_, 10 HEPES. Solutions were adjusted to pH 7.4 with CsOH. Pipettes were filled with intracellular solution, containing (in mM): 120 CsF, 20 CsCl, 10 NaCl, 10 HEPES, pH 7.4. All recordings were made using an EPC-9 patch-clamp amplifier (HEKA Elektronik, Lambrecht, Germany) digitized at 20 kHz via an ITC-16 interface (Instrutech, Great Neck, NY, USA). Voltage clamping and data acquisition were controlled using PatchMaster/FitMaster software (HEKA Elektronik, Lambrecht, Germany) running on an Apple iMac. Current was low-pass-filtered at 5 kHz. Leak subtraction was performed automatically by software using a P/4 procedure following the test pulse. Gigaohm seals were allowed to stabilize in the on-cell configuration for 1 min prior to establishing the whole-cell configuration. Series resistance was less than 5 MΩ for all recordings. Series resistance compensation up to 80% was used when necessary. All data were acquired at least 5 min after attaining the whole-cell configuration, and cells were allowed to incubate 5 min after drug application prior to data collection. Before each protocol, the membrane potential was hyperpolarized to -130 mV to insure complete removal of both fast-inactivation and slow-inactivation. Leakage and capacitive currents were subtracted with a P/4 protocol. All experiments were conducted at room temperature at 22°C.

### Analysis

Analysis and graphing were done using FitMaster software (HEKA Elektronik) and Igor Pro (Wavemetrics, LakeOswego, OR, USA) with statistical information derived using JMP statistical software version 11. All data acquisition and analysis programs were run on an Apple iMac (Apple Computer). A two-way analysis of variance (ANOVA) was used to compare the means responses [activation, peak current block, steady-state fast-inactivation (SSFI), late currents, and window currents] between channel variant, and compound. Channel variant, compound and the way interaction involving the two were considered to be fixed effects in the model. Channel variant had two levels (WT, ΔKPQ) and compound had six levels (0, 0.5, and 2.5 μM AMD and DES). *Post hoc* tests using the Tukey Kramer adjustment were used to compare mean responses between pairs of channel variant and/or compounds. A level of significance α = 0.05 was used in all overall *post hoc* tests, and effects with *p*-values less then 0.05 were considered to be statistically significant. All values reported are given as mean ± standard error of means for *n* cells.

### Activation Protocols

To determine the voltage dependence of activation, we measured the peak current amplitude at test pulse potentials ranging from -100 to +80 mV in increments of +10 mV for 20 ms. Channel conductance (G) was calculated from peak I_Na_:

$${\text{G}}_{\text{Na}}\;\text{=}\;{\text{I}}_{\text{Na}}{\text{/V-E}}_{\text{Na}}$$

where G_Na_ is conductance, I_Na_ is peak sodium current in response to the command potential V, and E_Na_ is the Nernst equilibrium potential. Calculated values for conductance were fit with the Boltzmann equation:

$${\text{G/G}}_{\text{max}}\;\text{=}\;\text{1/}(\text{1+exp}[{\text{-ze}}_{\text{0}}[{\text{V}}_{\text{m}}{\text{-V}}_{\text{1/2}}]\text{/kT}])$$

where G/G_max_ is normalized conductance amplitude, V_m_ is the command potential, z is the apparent valence, e_0_ is the elementary charge, V_1/2_ is the midpoint voltage, k is the Boltzmann constant, and T is temperature in °K.

### Steady-State Fast-Inactivation Protocols

The voltage-dependence of fast-inactivation was measured by preconditioning the channels to a hyperpolarizing potential of -130 mV and then eliciting pre-pulse potentials that ranged from -170 to +10 mV in increments of 10 mV for 500 ms, followed by a 10 ms test pulse during which the voltage was stepped to 0 mV. Normalized current amplitude as a function of voltage was fit using the Boltzmann equation:

$${\text{I/I}}_{\text{max}}\;\text{=}\;\text{1/}(\text{1+exp}({\text{-ze}}_{\text{0}}({\text{V}}_{\text{M}}{\text{-V}}_{\text{1/2}})\text{/kT})$$

where I_max_ is the maximum test pulse current amplitude.

### Window Current Measurements

Window currents were measured by maintaining the voltage at -130 mV for 1 s. Then, the voltage was ramped to 20 mV for 20 ms, at a rate of 0.3 mV/ms. The channels were then recovered from inactivation at the voltage of -130 mV for 20 ms.

### Drug Preparation

We sought to study the effects of AMD and DES on cardiac voltage-gated sodium channels using their therapeutic serum concentrations which, for both compounds, is 2.5 μM (Sheldon et al., 1989). However, xenobiotic concentrations decrease over time; therefore, to assess the effects of these reductions, our experiments were also conducted at 0.5 μM AMD and DES. To ensure no run-down of sodium currents is taking place, electrophysiological recordings were taken on average 3–5 min after AMD or DES perfusion, until current levels were stabilized.

### Simulations

Action potentials were simulated in OpenCell (Physiome Project) with the 2004 Hund-Rudy Canine ventricular cell model (Rate Dependence and Regulation of Action Potential and Calcium Transient in a Canine Cardiac Ventricular Cell Model, Hund and Rudy, 2004. http://models.cellml.org/exposure/f4b7120aa512c7f5e7a0664abcee3e8b/hund_rudy_2004_b.cellml/view 6.33 pm 12th January 2016 CellML author(s): Catherine Lloyd) (Hund and Rudy, 2004; Lloyd et al., 2008).

## Results

### Activation

**FIGURE 1:**
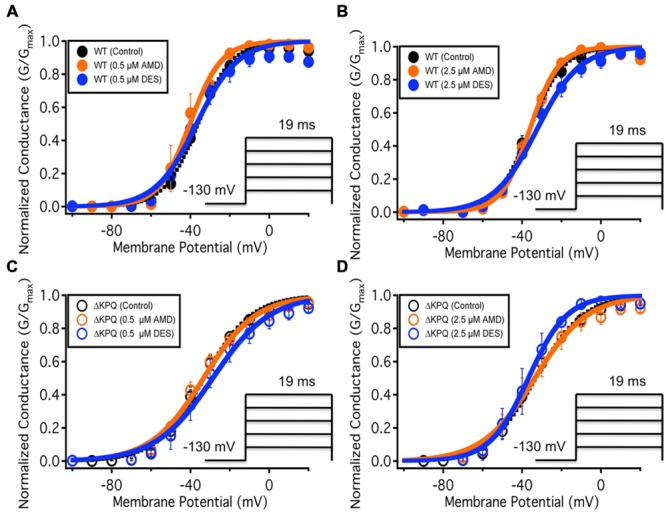
**(A–D)** Show the voltage dependence of activation as normalized conductance plotted against membrane potential. The black curves represent the WT Na_v_1.5 or ΔKPQ conductance with no drug perfusion, the orange curves represent Na_v_1.5 or ΔKPQ conductance after either 0.5 or 2.5 μM AMD perfusion, and the blue curves represent Na_v_1.5 or ΔKPQ conductance after either 0.5 or 2.5 μM DES perfusion.

**FIGURE 2:**
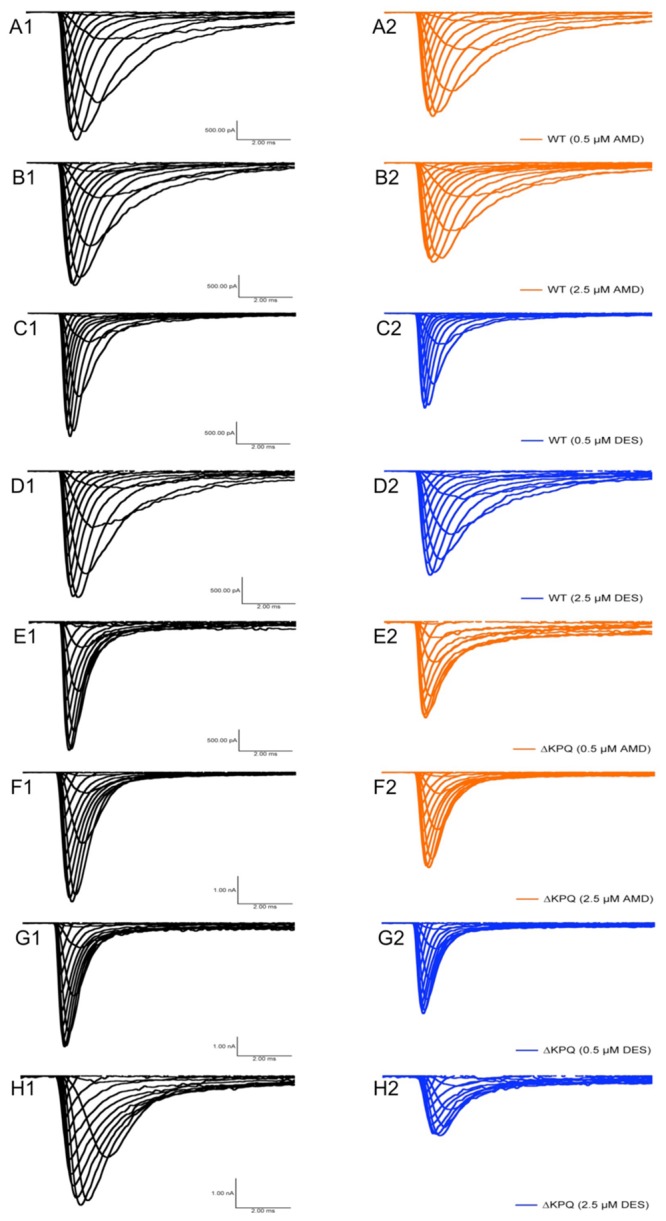
**Current amplitude plotted versus time duration.** The traces show the variation in current levels before and after AMD and DES perfusion.

We examined the effects of AMD and DES on activation in Na_V_1.5 and ΔKPQ channels. **Figure 1** shows normalized conductance plotted as a function of membrane potential under control solutions (black curves with filled circles), in AMD solutions (orange curves with filled circles), and DES solutions (blue curves with filled circles) in WT and ΔKPQ Na_V_1.5 channels. AMD and DES cause no significant effects on the voltage dependence of activation. There were no significant shifts in the midpoint or apparent valence at either 0.5 or 2.5 μM of AMD or DES in either Na_V_1.5 or ΔKPQ (*p* > 0.05; **Table 1**). Current traces from the eight experimental conditions (before and after AMD or DES perfusion) are shown in (**Figure 2**).

**Table 1:** Steady-state activation.

	GV – V_1/2_ (mV)	GV – z (slope)	*n*
**Wild-type**			
**Na_V_1.5**			
Control	-35.5 ± 1.63	3.79 ± 0.29	20
AMD 0.5 μM	-45.7 ± 4.42	3.91 ± 0.69	4
AMD 2.5 μM	-36.1 ± 1.45	3.54 ± 0.15	4
DES 0.5 μM	-35.5 ± 3.57	3.25 ± 0.54	7
DES 2.5 μM	-32.4 ± 1.87	2.67 ± 0.40	5
**ΔKPQ**			
**Na_V_1.5**			
Control	-33.6 ± 2.35	3.25 ± 0.34	20
AMD 0.5 μM	-34.8 ± 2.75	2.29 ± 0.24	5
AMD 2.5 μM	-35.1 ± 4.61	2.63 ± 0.26	6
DES 0.5 μM	-31.1 ± 5.34	2.26 ± 0.16	4
DES 2.5 μM	-31.1 ± 3.10	2.66 ± 0.53	4

### Peak Current Block

Amiodarone is a sodium channel blocker. To determine whether there is a difference between AMD and DES in their ability to block INa, we compared the peak current in each channel variant before and after the perfusion of either compound at one of the noted concentrations. Although current block was seen across all conditions, there was a significant mutant effect, indicating that both the mother compound, AMD, and its metabolite, DES, block ΔKPQ more than they block Na_V_1.5 (*p* < 0.05; **Table 2**). The largest block was observed in ΔKPQ mutants at 2.5 μM DES (**Table 2**).

**Table 2:** Peak I_Na_ block.

	Peak I_Na_ block	*n*
**Wild-type**		
**zNa_V_1.5**		
AMD 0.5 μM	0.05 ± 0.11	3
AMD 2.5 μM	0.14 ± 0.12	6
DES 0.5 μM	0.32 ± 0.05	7
DES 2.5 μM	0.12 ± 0.13	6
**ΔKPQ**		
**Na_V_1.5**		
AMD 0.5 μM	0.39 ± 0.12	6
AMD 2.5 μM	0.38 ± 0.28	4
DES 0.5 μM	0.28 ± 0.04	4
DES 2.5 μM	0.55 ± 0.12	6

### Steady-State Fast-Inactivation

**FIGURE 3:**
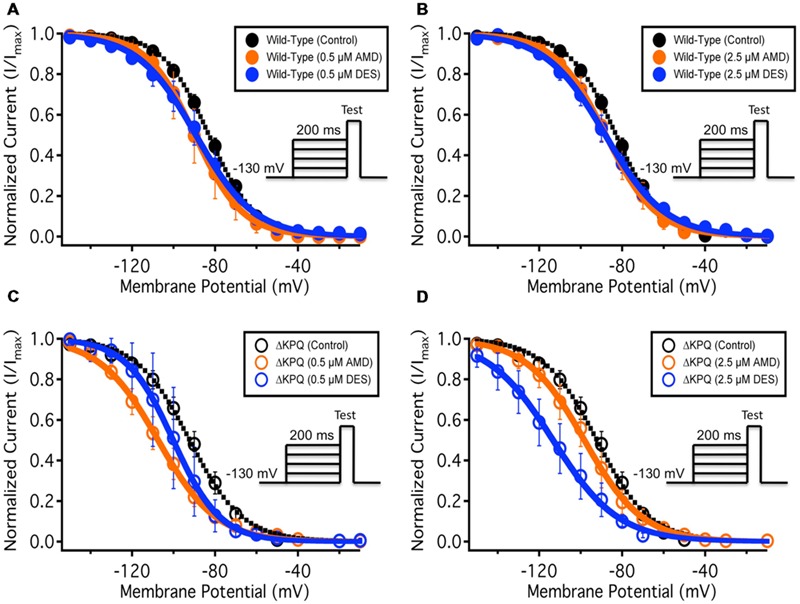
**(A–D)** Show the voltage dependence of steady-state fast-inactivation as normalized current plotted against membrane potential. The black curves represent the WT Na_v_1.5 or ΔKPQ SSFI with no drug perfusion, the orange curves represent Na_v_1.5 or ΔKPQ SSFI after either 0.5 or 2.5 μM AMD perfusion, and the blue curves represent Na_v_1.5 or ΔKPQ SSFI after either 0.5 or 2.5 μM DES perfusion.

**FIGURE 4:**
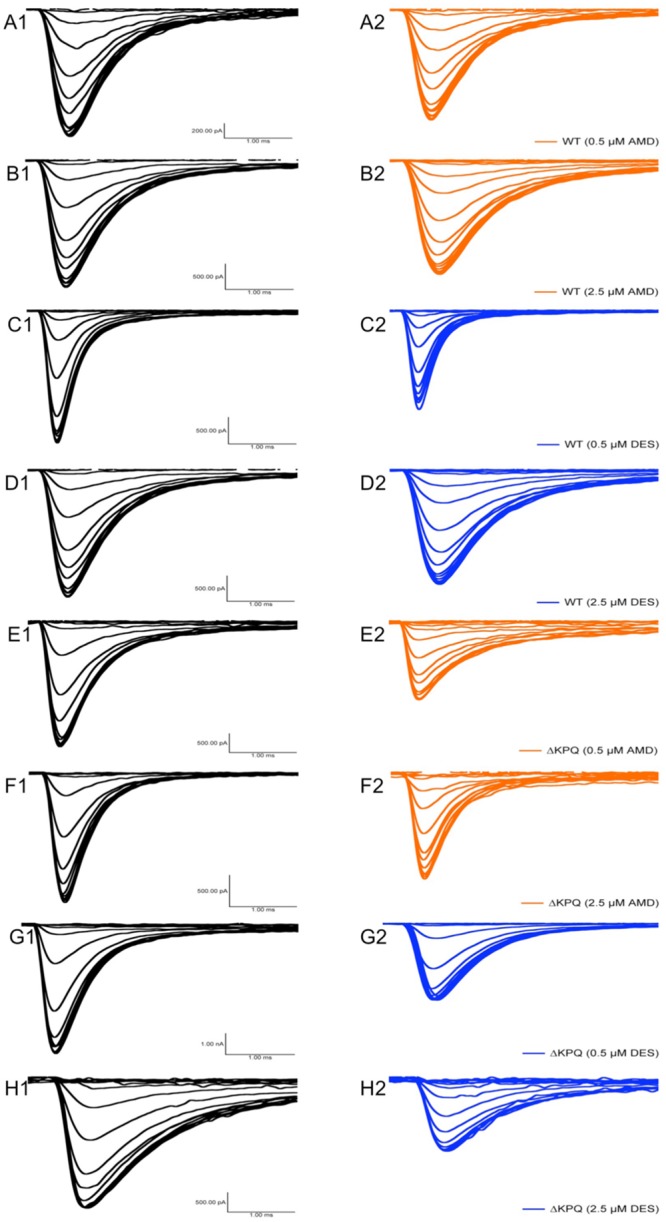
**Fast-inactivation traces.** Traces show the variation in current levels before and after AMD and DES perfusion.

To quantify the effects of each compound at 0.5 and 2.5 μM on fast-inactivation, we compared the apparent valence (z) and midpoint (V_1/2_) from Boltzmann fits to SSFI data for Na_V_1.5 (**Figures 3A,B**) and ΔKPQ (**Figures 3C,D**). Normalized current amplitudes were plotted as a function of pre-pulse potential (**Figure 3**). The apparent valence of SSFI of ΔKPQ (2.79 ± 0.15) was greater than that of WT Na_V_1.5 (2.68 ± 0.10). AMD and DES at 0.5 and 2.5 μM significantly reduced the apparent valence of SSFI in both Na_V_1.5 and ΔKPQ (*p* < 0.05; **Table 3**). The V_1/2_ of ΔKPQ was significantly hyperpolarized compared to WT (*p* < 0.05); however, there were no significant shifts in the V_1/2_ of either channel variant with AMD or DES at either concentration (*p* > 0.05; **Table 3**). Representative traces are shown in (**Figure 4**).

**Table 3:** Steady-state fast-inactivation.

	SSFI – V_1/2_ (mV)	SSFI – z (slope)	*n*
**Wild-type**			
**Na_V_1.5**			
Control	-83.0 ± 1.82	-2.68 ± 0.10	20
AMD 0.5 μM	-89.1 ± 6.04	-2.81 ± 0.33	4
AMD 2.5 μM	-87.4 ± 3.79	-2.36 ± 0.15	7
DES 0.5 μM	-89.5 ± 4.38	-2.19 ± 0.12	7
DES 2.5 μM	-88.4 ± 3.00	-1.89 ± 0.14	5
**ΔKPQ**			
**Na_V_1.5**			
Control	-92.3 ± 3.06	-2.79 ± 0.15	20
AMD 0.5 μM	-107.3 ± 3.69	-2.08 ± 0.16	6
AMD 2.5 μM	-94.9 ± 5.21	-2.22 ± 0.19	5
DES 0.5 μM	-100.3 ± 4.86	-2.74 ± 0.34	4
DES 2.5 μM	-107.8 ± 5.24	-2.38 ± 0.21	5

### Late Sodium Currents

**FIGURE 5:**
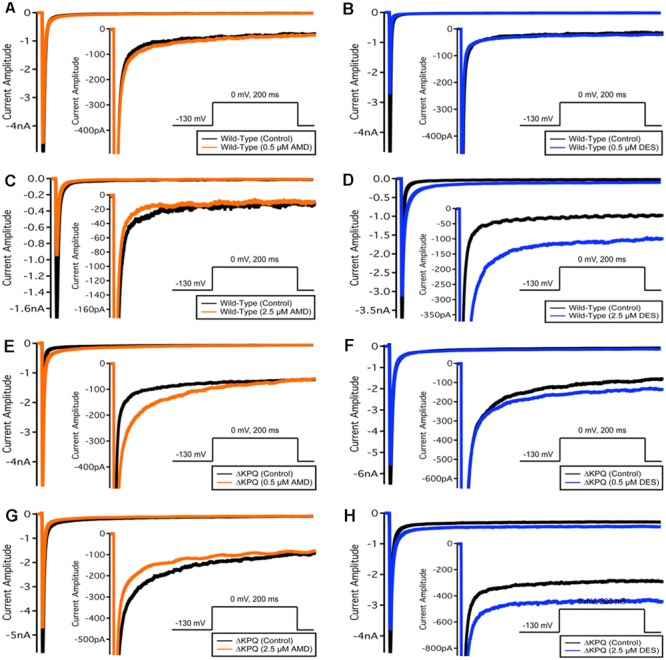
**Representative normalized current traces of INa_L_. (A–H)** Each panel shows the INa_L_ in Na_V_1.5 or ΔKPQ before and after the perfusion of either 0.5 or 2.5 μM AMD or DES. Black curves represent control (0 μM drug solution), orange curves represent AMD at either 0.5 or 2.5 μM, and blue curves represent DES at either 0.5 or 2.5 μM.

**FIGURE 6:**
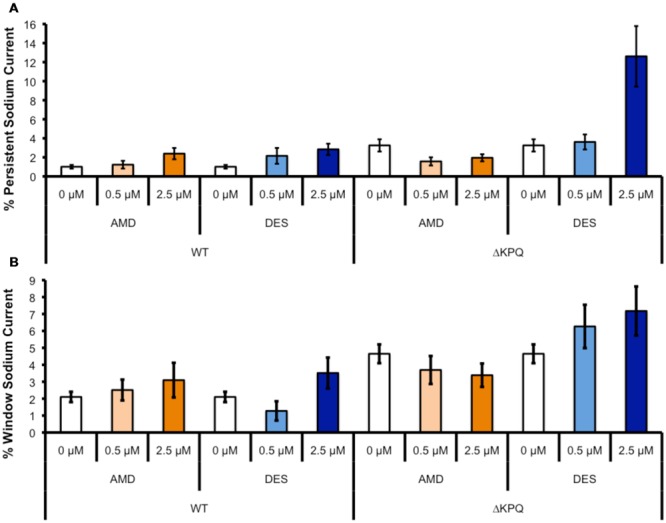
**(A)** Shows the percentage of persistent sodium currents in either WT Na_v_1.5 or ΔKPQ channels after the perfusion of 0, 0.5, or 2.5 μM AMD (light or dark orange) or DES (light or dark blue). **(B)** Shows the percentage of window sodium currents in either WT Na_v_1.5 or ΔKPQ channels after the perfusion of 0, 0.5, or 2.5 μM AMD (light or dark orange) or DES (light or dark blue).

The presence of I_Na,L_ is an indicator of destabilized fast-inactivation. We show representative normalized current traces of I_Na,L_ for the two channel variants across all conditions (**Figure 5**). Although, both compounds appear to have statistically significant effects on elevating I_Na,L_ levels (*p* < 0.01), the most prominent I_Na,L_ increase was seen upon the perfusion of 2.5 μM DES on ΔKPQ (**Figure 6A**; **Table 4**). Therefore, therapeutic levels of DES potently further destabilize fast-inactivation in ΔKPQ mutants.

**Table 4:** Persistent I_Na_.

	Persistent I_Na_	*n*
**Wild-type**		
**Na_V_1.5**		
Control	0.88 ± 0.16	23
AMD 0.5 μM	1.23 ± 0.41	6
AMD 2.5 μM	2.39 ± 0.59	6
DES 0.5 μM	2.16 ± 0.83	6
DES 2.5 μM	2.84 ± 0.59	5
**ΔKPQ**		
**Na_V_1.5**		
Control	2.79 ± 0.55	18
AMD 0.5 μM	2.89 ± 1.35	6
AMD 2.5 μM	2.62 ± 0.73	6
DES 0.5 μM	3.62 ± 0.79	5
DES 2.5 μM	12.6 ± 3.18	4

### Sodium Window Currents

**FIGURE 7:**
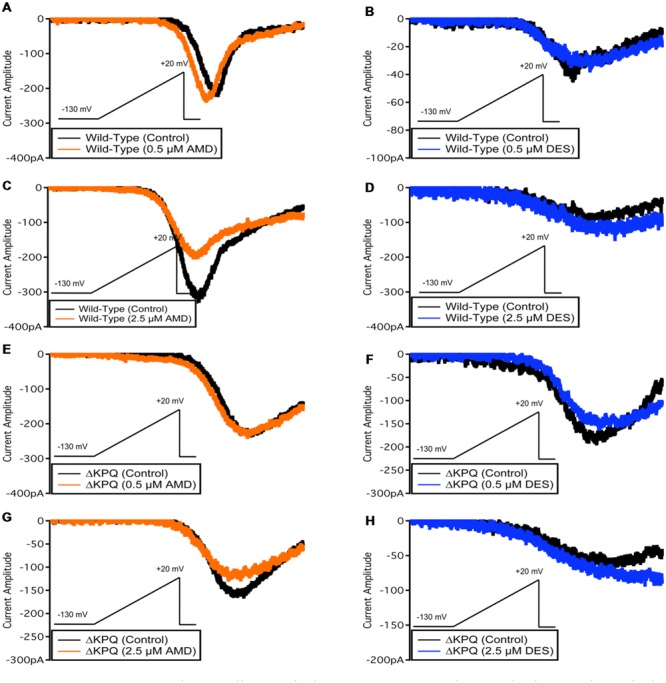
**Representative sodium window currents. (A–H)** Each panel shows the window currents in Na_V_1.5 or ΔKPQ before and after the perfusion of either 0.5 or 2.5 μM AMD or DES. Black curves represent control (0 μM drug solution), orange curves represent AMD at either 0.5 or 2.5 μM, and blue curves represent DES at either 0.5 or 2.5 μM.

Changes in both steady-state activation and fast-inactivation can lead to differences in the window current. Representative normalized traces of sodium window currents from all conditions are shown in (**Figure 7**). Similar to I_Na,L_, there was an significant compound effect on the sodium window currents (*p* < 0.05), where the greatest effect was seen in ΔKPQ at 2.5 μM DES (**Figure 6B**; **Table 5**).

**Table 5:** Ramp I_Na_.

	Ramp I_Na_	*n*
**Wild-type**		
**Na_V_1.5**		
Control	1.77 ± 0.25	19
AMD 0.5 μM	2.51 ± 0.62	5
AMD 2.5 μM	3.10 ± 1.02	4
DES 0.5 μM	1.28 ± 0.57	6
DES 2.5 μM	3.52 ± 0.91	4
**ΔKPQ**		
**Na_V_1.5**		
Control	4.30 ± 0.47	22
AMD 0.5 μM	1.84 ± 0.82	5
AMD 2.5 μM	3.39 ± 0.69	5
DES 0.5 μM	6.27 ± 1.28	6
DES 2.5 μM	7.18 ± 1.45	5

### Action Potential Modeling

**FIGURE 8:**
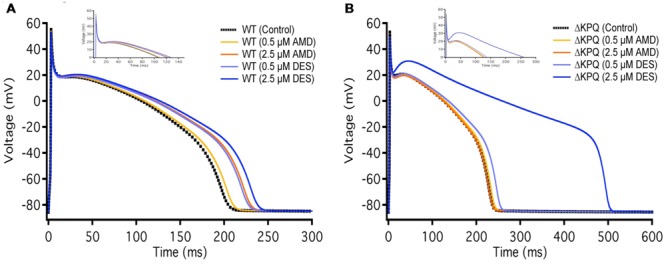
**Action potential model simulation. (A)** Shows the simulations belonging to WT Na_v_1.5, where the black curve represents control, light and dark orange curves represent AMD perfusion at 0.5 and 2.5 μM, respectively, and light and dark blue curves represent DES perfusion at 0.5 and 2.5 μM, respectively. **(B)** Shows the simulations belonging to ΔKPQ, where the black curve represents control, light and dark orange curves represent AMD perfusion at 0.5 and 2.5 μM, respectively, and light and dark blue curves represent DES perfusion at 0.5 and 2.5 μM, respectively.

The duration of action potentials plays a fundamental role in the functioning of cardiac myocytes. The alteration of any contributors in action potential generation and maintenance could lead to detrimental effects such as life-threatening arrhythmias. In order to determine whether the observed compound-mediated fast-inactivation destabilization can lead to pro-arrhythmogenicity, we used the Hund-Rudy Canine model to simulate a cardiac action potential (**Figure 8**). The simulation results indicate that the extent of action potential prolongation is consistent with our experimental data showing compound-mediated exacerbation in I_Na,L_. As expected, the APD of ΔKPQ was longer than Na_V_1.5, hence the LQT phenotype. Furthermore, the model suggests that the biggest pro-arrhythmogenic effect occurs at 2.5 μM DES in ΔKPQ mutants (**Figure 8**; **Table 6**).

**Table 6:** Action potential duration.

	Time (ms)	Voltage (mV)
**Wild-type**		
**Na_V_1.5**		
Control	198.3	-70.0
AMD 0.5 μM	204.4	-70.0
AMD 2.5 μM	224.3	-70.0
DES 0.5 μM	221.4	-70.0
DES 2.5 μM	231.9	-70.0
**ΔKPQ**		
**Na_V_1.5**		
Control	230.4	-70.0
AMD 0.5 μM	235.2	-70.0
AMD 2.5 μM	230.2	-70.0
DES 0.5 μM	247.8	-70.0
DES 2.5 μM	496.1	-70.0

## Discussion

Since its development, the use of AMD as a therapeutic has fluctuated due to occasional reports of toxicity in some individuals. These safety issues have resulted in AMD being regarded as a dangerous drug; however, the efficacy of this drug against certain conditions, such as atrial fibrillation, is well-established.

We performed the first detailed study on the effects of AMD on cardiac voltage-gated sodium channels. We included DES, a physiologically active metabolite of AMD, into our study, and compared the effects of AMD and DES on biophysical properties in WT Na_V_1.5 and ΔKPQ, a channel variant with an exaggerated late current. Neither AMD nor DES affect the voltage dependence of activation or SSFI. However, a decrease in the apparent valence in SSFI of both channels suggests that the compounds reduce the Na_V_1.5 and ΔKPQ charge sensitivities.

Although AMD has been recognized as a sodium channel blocker, DES has not been fully characterized with respect to its effects on sodium channels. Here, we show that DES also blocks sodium channels. Interestingly, both compounds have a significant preference in blocking ΔKPQ compared to WT Na_V_1.5.

Our results indicate that DES significantly increases both I_Na,L_ and window currents in ΔKPQ mutants. Given that the presence of I_Na,L_ is a manifestation of the failure to fast-inactivate, we conclude that DES further disrupts ΔKPQ fast-inactivation.

In an earlier study, Maltsev et al. (2001) performed electrophysiological studies on cardiac cells isolated from failing human hearts. They concluded that AMD blocks late sodium currents in these cells. However, within the same study they mentioned that the interpretation of their results is rather complicated due to the I_Na,L_ density variations in the cardiomyocytes from different patients (Maltsev et al., 2001). These variations point to the differences in the genetic background present in isolated myocytes, which may have led to the observed results in that study. In order to reduce the confounding effects of the rather complex genetic background in human myocytes, we performed our studies in the simpler CHO cells. This allowed us to understand the biophysics of the interactions between AMD/DES and Na_V_1.5/ΔKPQ on a fundamental level, and then apply that information to a generic myocyte action potential model using computer simulations.

In another study conducted by Wu et al. (2008) attempting to characterize AMD, sea anemone toxin II (ATX-II) was used to induce I_Na,L_ in Na_V_1.5. This study concluded that ATX-II-mediated I_Na,L_ is blocked by AMD (Wu et al., 2008). These results led us to initially hypothesize that AMD and DES would block both I_Na,L_ and I_Na,P_; however, ATX-II is also a chemical agent that interacts with sodium channels. To eliminate inter-compound interactions between ATX-II and AMD or DES, we used ΔKPQ. The pro-arrhythmic effects of these compounds on I_Na,L_ were, particularly notable. Mutational studies in Na_V_1.2 indicate that ATX-II tends to interact close to the glutamic acid residue at position 1613 on the DIVS3-S4 extracellular loop (Rogers et al., 1996). Considering AMD and DES were perfused on the extracellular side of the channels in our experiments, along with previous findings in Na_V_1.2, our contradictory results to Wu et al. (2008) seem justified. We predict that the mode of action of AMD may involve interactions with DIVS3-S4 of the sodium channel. This would indicate that AMD outcompetes ATX-II for binding sites on the sodium channel. This hypothesis may explain the decrease in I_Na,L_ in ATX-II-treated channels; however, this hypothesis needs to be tested in future studies.

It is well-known that the prolongation of the QT interval on the electrocardiogram is a prominent risk factor for arrhythmias, which may lead to sudden death. There are many pathophysiological mechanisms that may underlie this prolongation; however, those that increase the APD are perhaps most common. Sodium channels are a key contributor to action potential generation and propagation (Catterall et al., 2005); therefore, disrupting these channels’ ability to fast-inactivate could result in the elongation of the APD, which could prolong the QT interval leading to arrhythmogenicity. According to our simulation results, AMD and DES indeed increase APD in myocytes. Thus, we conclude that these compounds could contribute to pro- arrhythmogenicity.

Despite the possibility of having pro-arrhythmic effects, our findings suggest that AMD and DES can also have an anti-arrhythmic effect in that they block I_Na,P._ This mode of action is not unique to these compounds. Similar to AMD and DES, Wang et al. (1990) have shown that a compound called DPI 201-106 slows cardiac sodium channel inactivation, followed by blocking peak inward sodium currents.

*N*-desethylamiodarone seems to be a more potent pro-arrhythmic agent than the AMD from which it is metabolized. Since the LQT diagnosis is identified with an abnormally long QT interval, the use of AMD in a patient with this condition may be lethal. Therefore, our findings are a further validation for caution in the use of AMD in LQT patients, and specifically in those with the ΔKPQ mutation. Moreover, although AMD and DES have similar chemical structures, as we have shown, their effects on ionic sodium currents are not identical. Thus, we predict further characterization of AMD’s long list of metabolites may uncover substantial clinically relevant information.

## Author Contributions

M-RG and MA collected, assembled, analyzed, and interpreted the data. PCR conceived the experiments and revised the manuscript critically for important intellectual content.

## Conflict of Interest Statement

The authors declare that the research was conducted in the absence of any commercial or financial relationships that could be construed as a potential conflict of interest.
